# The elephant in the room: the stressful psychological effects of COVID-19 pandemic in mental healthcare workers

**DOI:** 10.1192/j.eurpsy.2022.779

**Published:** 2022-09-01

**Authors:** A. Minelli, S. Barlati, M. Vezzoli, S. Carletto, C. Isabello, A. Vita

**Affiliations:** 1 University of Brescia, Department Of Molecular And Translational Medicine, Brescia, Italy; 2 University of Brescia, Department Of Clinical And Experimental Sciences, Brescia, Italy; 3 University of Torino, Department Of Neuroscience “rita Levi Montalcini”, Torino, Italy; 4 ASL TO3, Mental Health Service Of Susa, Torino, Italy

**Keywords:** psychological distress, Covid-19, mental healthcare workers, resilience/vulnerability

## Abstract

**Introduction:**

Despite the large amount of research concerning the impact of COVID-19 on health care workers, to date few targeted MHWs. Moreover, none has investigated the vulnerability due to exposure to previous traumatic events among health care workers.

**Objectives:**

This study aimed to investigate the psychological distress in MHWs after the first lockdown imposed by the COVID-19 pandemic in the more impacted regions of the North of Italy, to understand which COVID-19, sociodemographic and professional variables as well as previous stressful life experiences, could have had greater negative effects.

**Methods:**

The online survey occurred from 28-June to 10-August 2020. This included questions regarding sociodemographic factors, professional information, COVID-19 exposure. Moreover, three validated self-report questionnaires were administered: Life Events Checklist for DSM-5 (LEC-5), Impact of Event Scale-Revised (IES-R), Depression Anxiety Stress Scales-21 (DASS-21).

**Results:**

271 MHWs completed the survey. At least 20% had elevated levels of psychological distress with post-traumatic symptoms. Stratifying for professional roles, the nurses resulted the most affected, with significantly higher scores in terms of intrusive thoughts, hyperarousal and avoidance behaviors. Several variables affected psychological distress in MHWs, but stronger effects were done by age, professional roles, increased workload and worst working environment during COVID-19 pandemic, to had experienced the separation of family members, but also had experienced during their life of a severe human suffering (physical and/or psychological) on oneself or on a loved one.

**Conclusions:**

Our data underlying the importance of recent but also previous severe stressful events as risk factors to develop post-traumatic symptoms reducing the resilience of the subjects investigated.

**Disclosure:**

No significant relationships.

